# Supplementary material for: Composition change-driven texturing and doping in solution-processed SnSe thermoelectric thin films
Source: Nat Commun. 2019 Feb 20;10:864. doi: 10.1038/s41467-019-08883-x (PMC6382880; doi:10.1038/s41467-019-08883-x)
Supplement: Supplementary file 1 — Supplementary Information [file 41467_2019_8883_MOESM1_ESM.pdf]

Composition change-driven texturing and doping in solution-processed SnSe thermoelectric thin films

Heo, et al.

* Correspondence to: hshin@kriss.re.kr (H.S.) and jsson@unist.ac.kr (J.S.S.)

This supplement contains

Supplementary Figures 1-13

Supplementary Tables 1-2

Supplementary References

Supplementary Figures

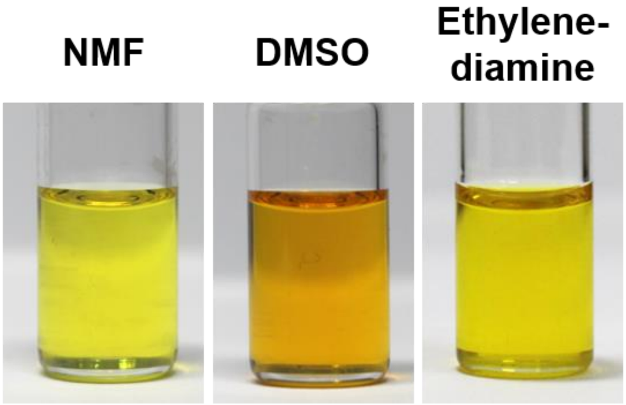

Supplementary Fig. 1 | Photographs of the SnSe ink solution dispersed in various solvents.

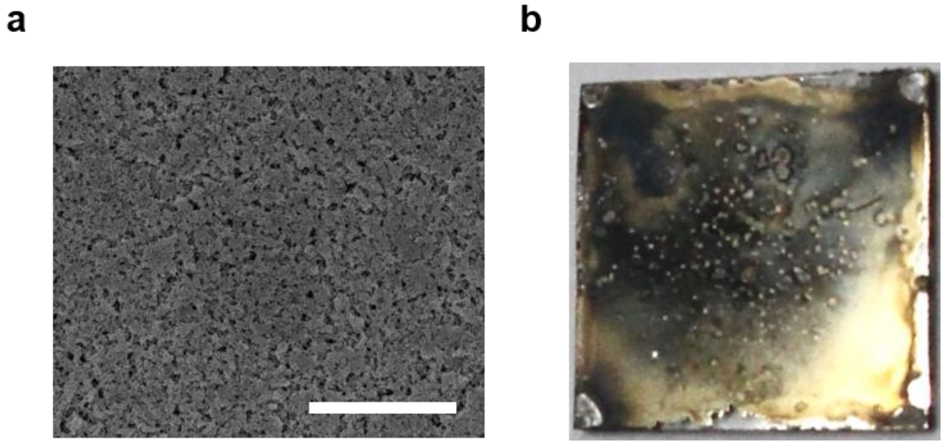

Supplementary Fig. 2 | The SnSe thin film fabricated with as-synthesized SnSe ink solution. a, SEM image of the SnSe thin film. The sample exhibits particulate morphology with multiple pinholes and pores. Scale bar, 5 μm. **b**, The photograph of the corresponding thin film.

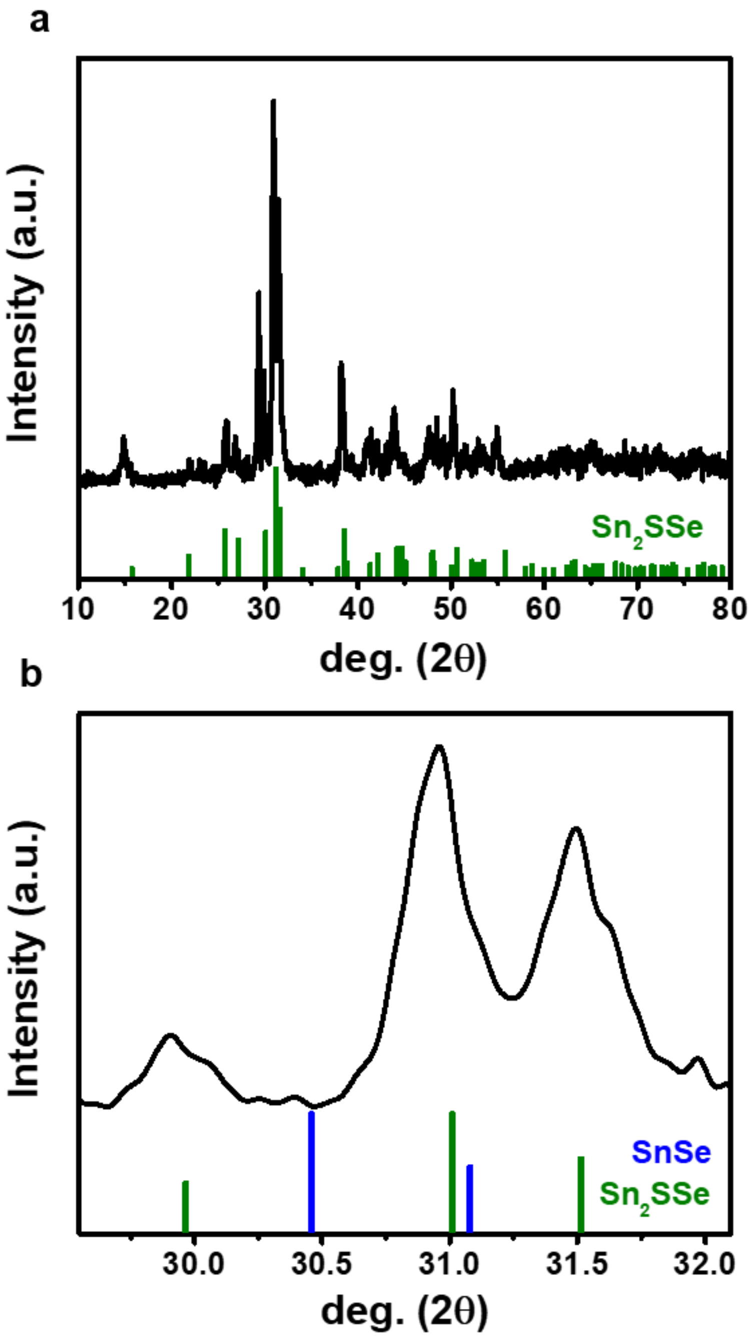

Supplementary Fig. 3 | XRD patterns of the thin film fabricated with as-synthesized solution. a,b XRD pattern of SnSe thin film corresponds to the pattern of orthorhombic Sn_2SSe phase. The vertical lines in (a) and (b) indicate the orthorhombic Sn_2SSe (green, JCPDS 71-5582). and the orthorhombic SnSe phase (blue, JCPDS 32-1382) respectively.

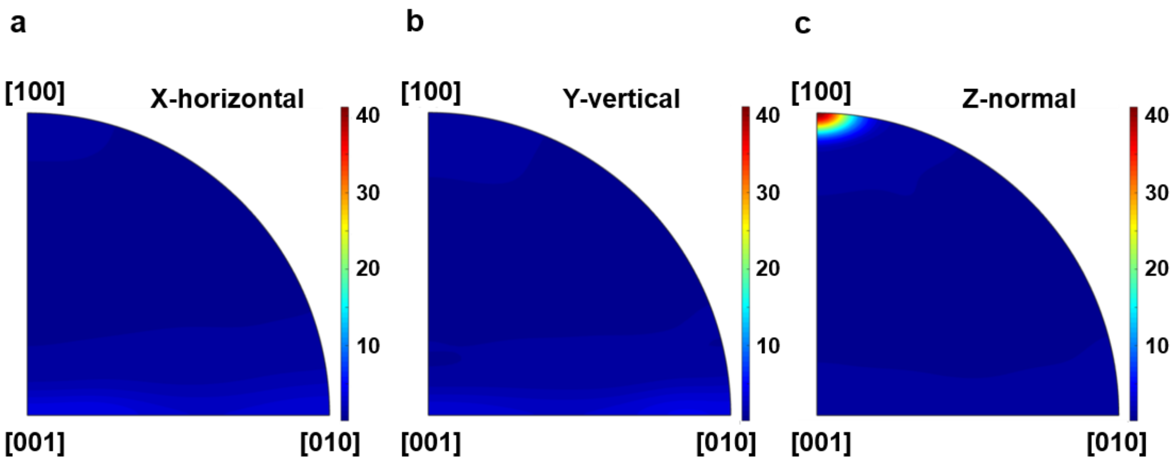

Supplementary Fig. 4 | Inverse pole figures of the thin film fabricated using the purified solution. Inverse pole figures in (**a**) horizontal direction (X), (**b**) vertical direction (Y), and (**c**) normal direction (Z) of the SnSe thin fabricated with purified precursor solution. The strong intensity at [100] direction in the inverse pole figure in normal direction demonstrates the highly oriented texture of the SnSe thin film.

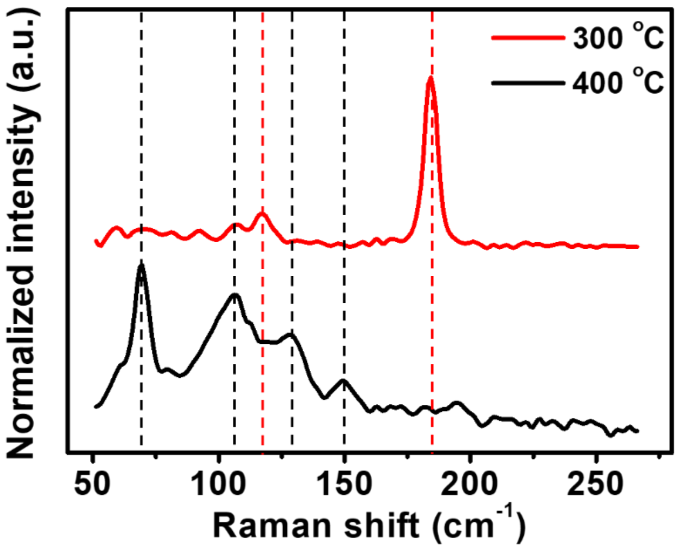

Supplementary Fig. 5 | Raman spectra of the SnSe thin films annealed at 300 ºC and 400 ºC.

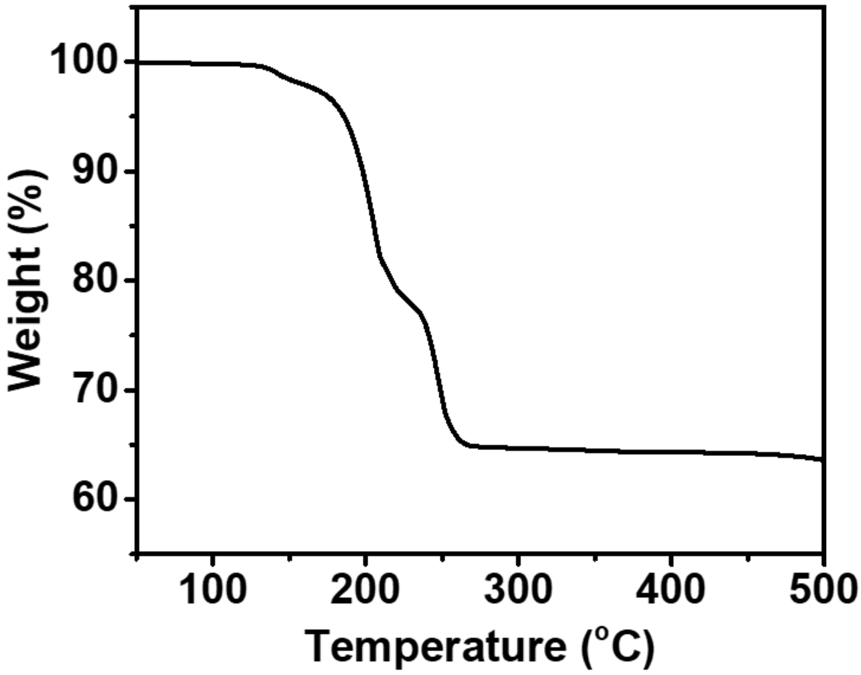

Supplementary Fig. 6 | Thermogravimetric analysis (TGA) analysis of the SnSe ChaM precursor. The TGA analysis of the SnSe ChaM shows the sharp decline of weight at ~187 ^{o}C and ~237 ^{o}C, and continuous weight loss above 300 ^{o}C.

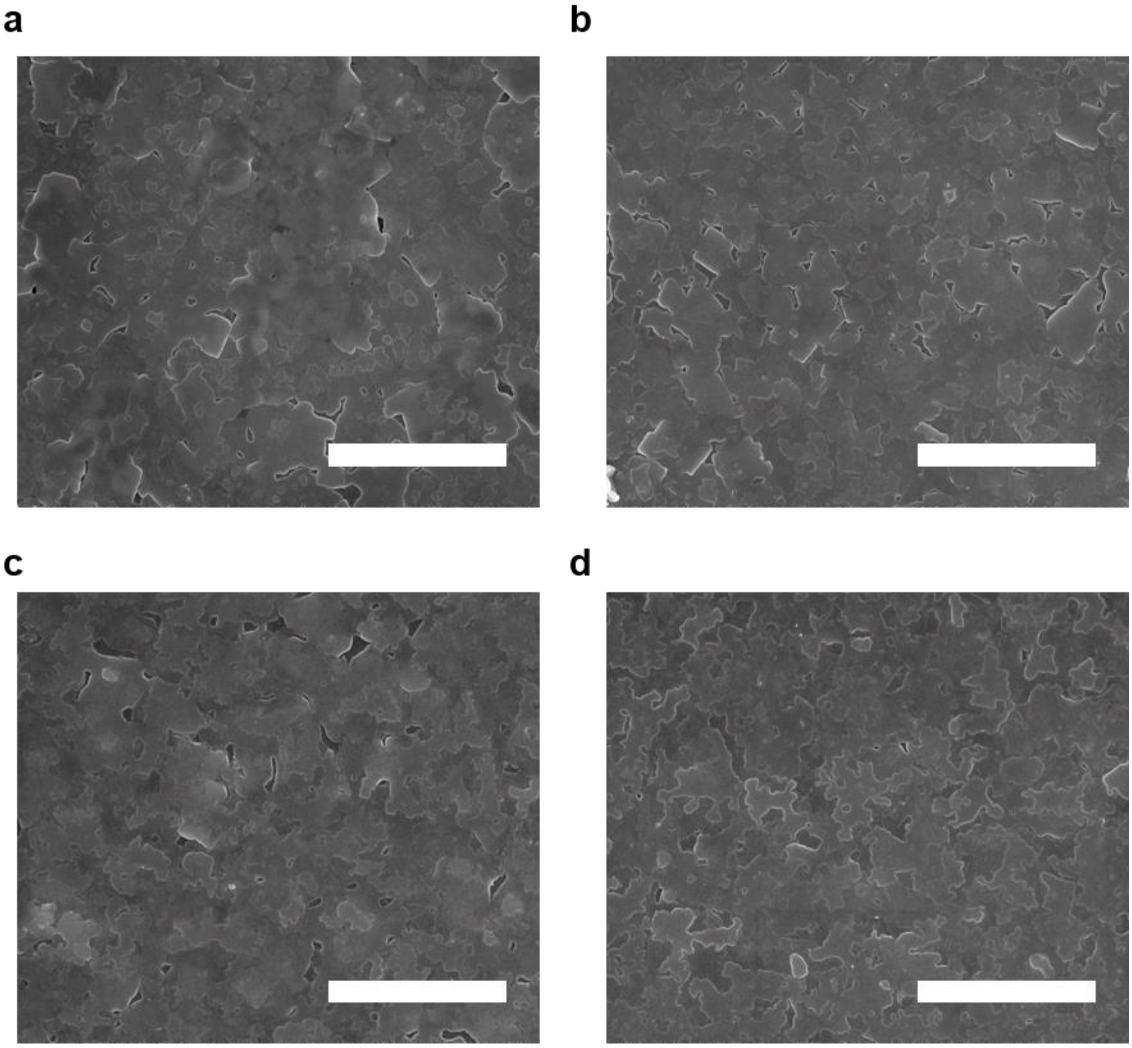

Supplementary Fig. 7 | SEM images of the SnSe thin films with different dwelling time. All SEM images of SnSe thin films treated at 400 ^{o}C for (**a**) 1 min, (**b**) 5 min, (**c**) 9 min, and (**d**) 13 min show the plate-like morphology in microstructures regardless of heat treatment time. Scale bar, 5 μm.

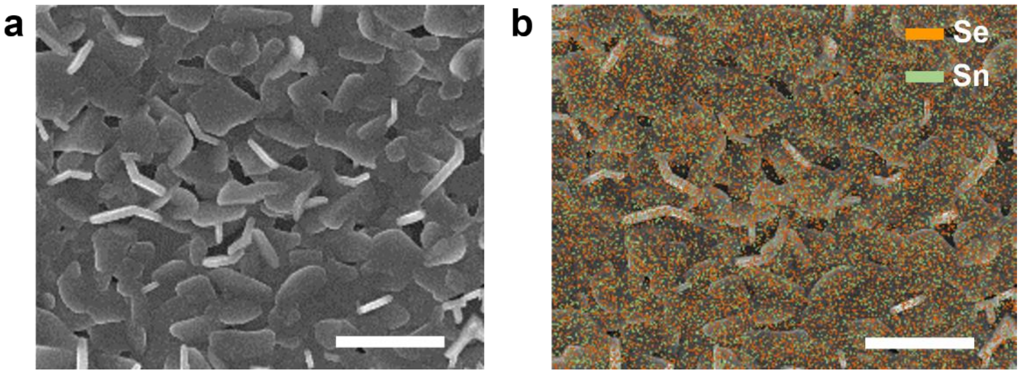

Supplementary Fig. 8 | SEM and EDS mapping image of SnSe thin film heat treated over 30 min. a,b. The SEM image shows microstructure deformation caused by severe Se evaporation after long duration at 400 ^{o}C. Scale bar, 2 μm.

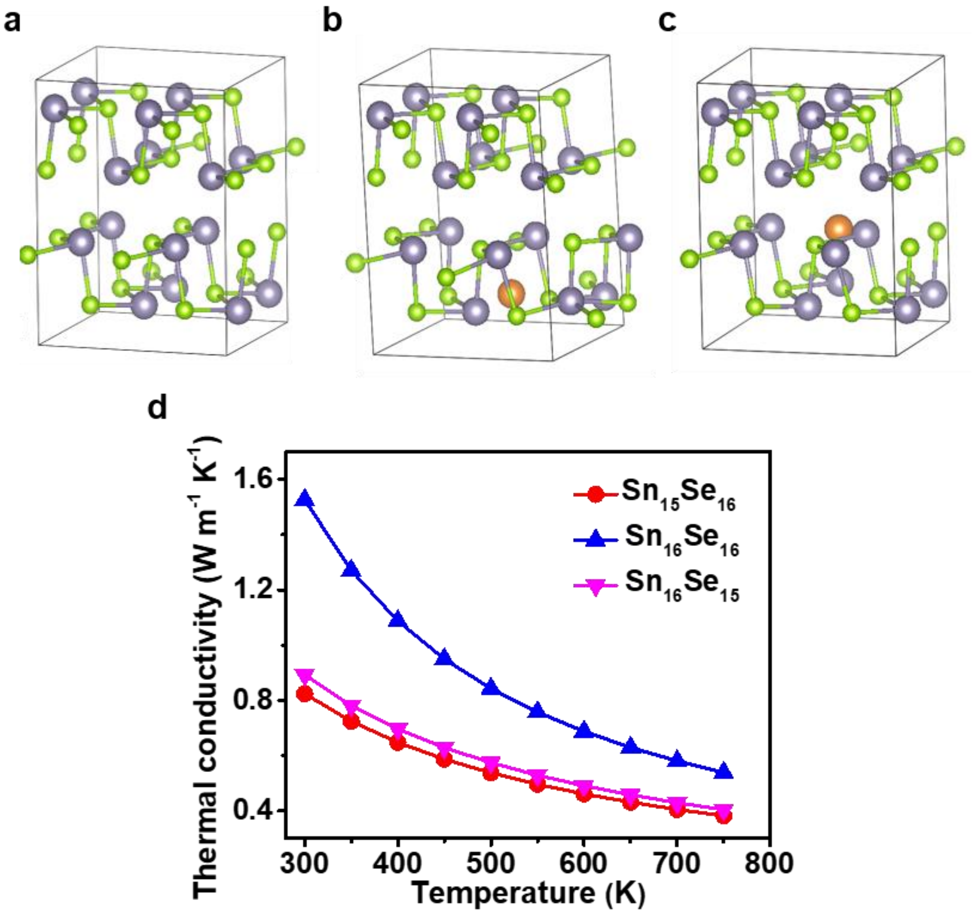

Supplementary Fig. 9 | First-principle calculation for thermal conductivities of SnSe. The unit cell structures of (**a**) $Sn_{16}Se_{16}$, (**b**) $Sn_{15}Se_{16}$, and (**c**) $Sn_{16}Se_{15}$ for calculation. Gray, green, and orange spheres in a-c represents Sn, Se, and vacancy, respectively. **d**, Calculated thermal conductivities for SnSe with Sn:Se atomic ratio of 15:16, 16:16, and 16:15.

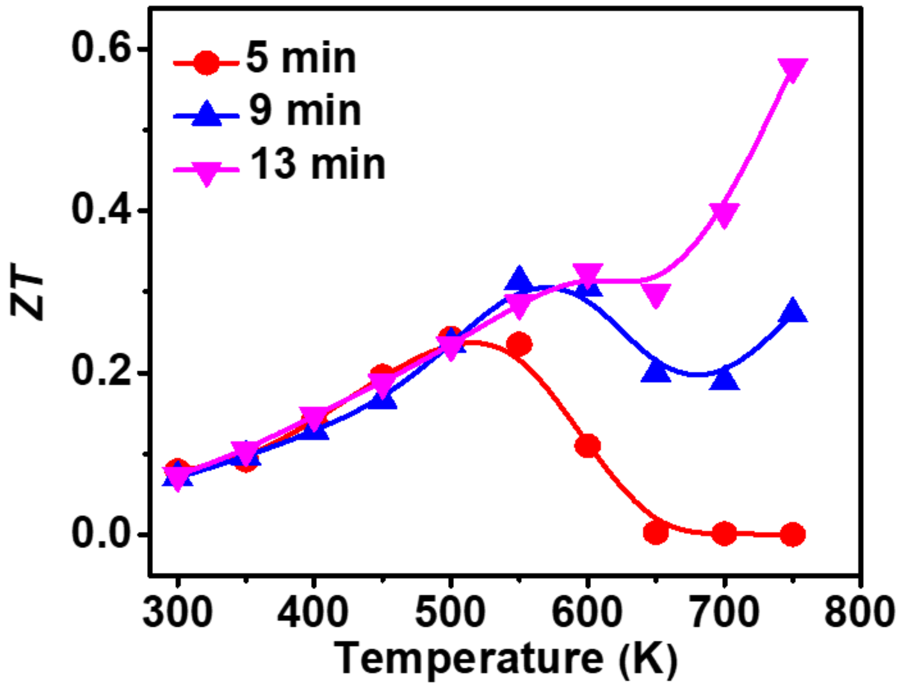

Supplementary Fig. 10 | Estimated ZT values for the SnSe thin films. Stars indicate data points obtained with SnSe single crystals in *a*-(black), *b*-(red), and *c*-(blue) axes[1].

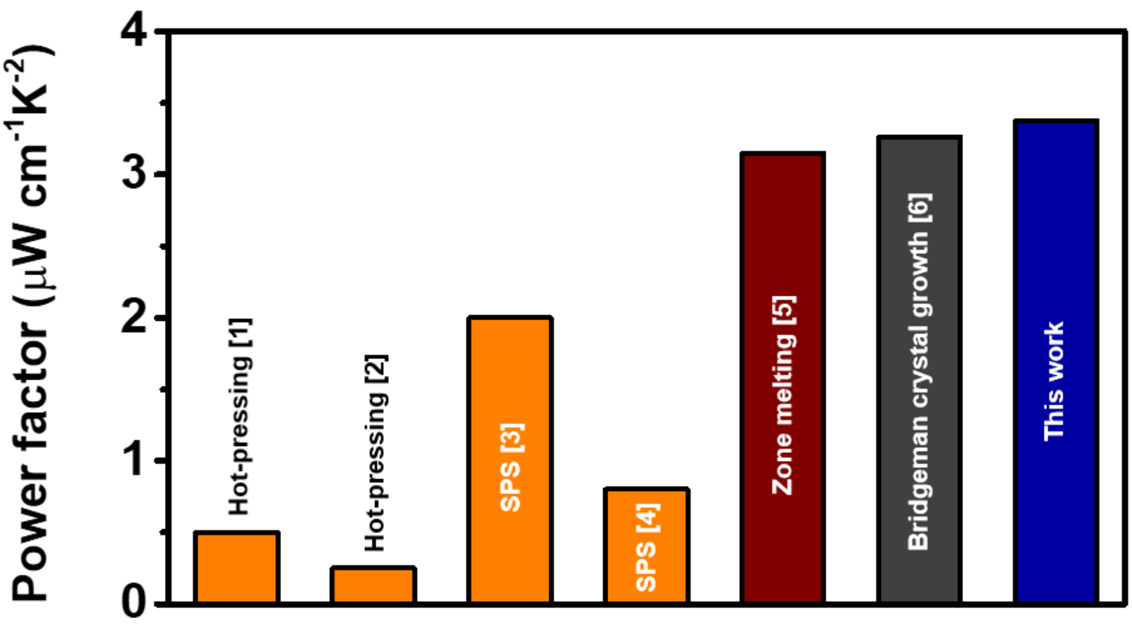

Supplementary Fig. 11 | Comparison of the power factors at room temperature. Power factors of polycrystalline bulk SnSe prepared by hot-pressing[2,3], spark plasma sintering (SPS)[4,5], and zone melting[6], and a power factor of single crystalline bulk SnSe prepared by Bridgeman crystal growth method[1] are compared with that of the currently developed highly textured SnSe thin film.

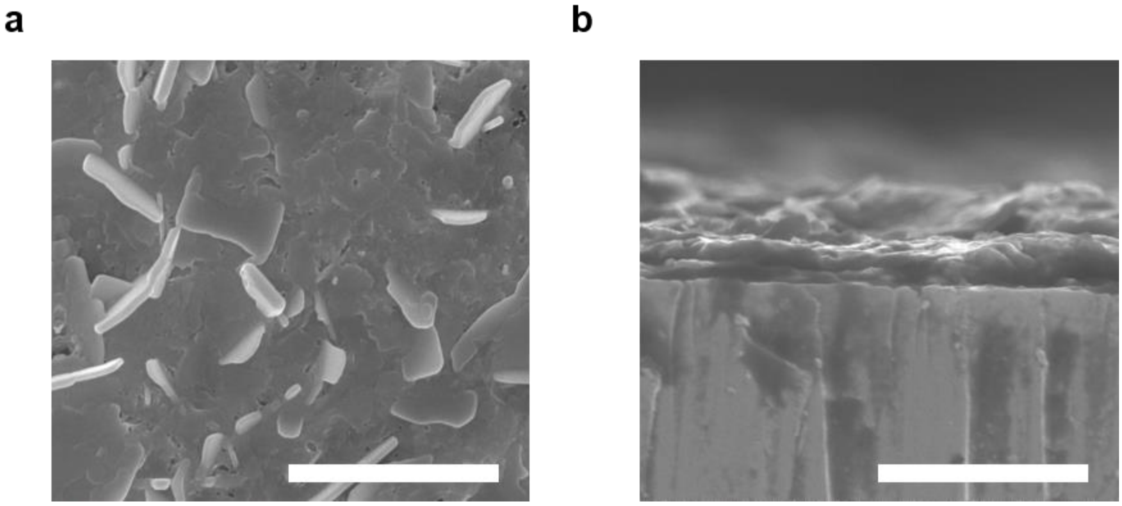

Supplementary Fig. 12 | Microstructure of spray-coated SnSe thin film. a, SEM image of SnSe thin film fabricated via spray-coating. The sample shows the plate-like morphology in microstructure. Scale bar, 3 μm. **b**, Cross-sectional SEM image of same sample. Scale bar, 3 μm.

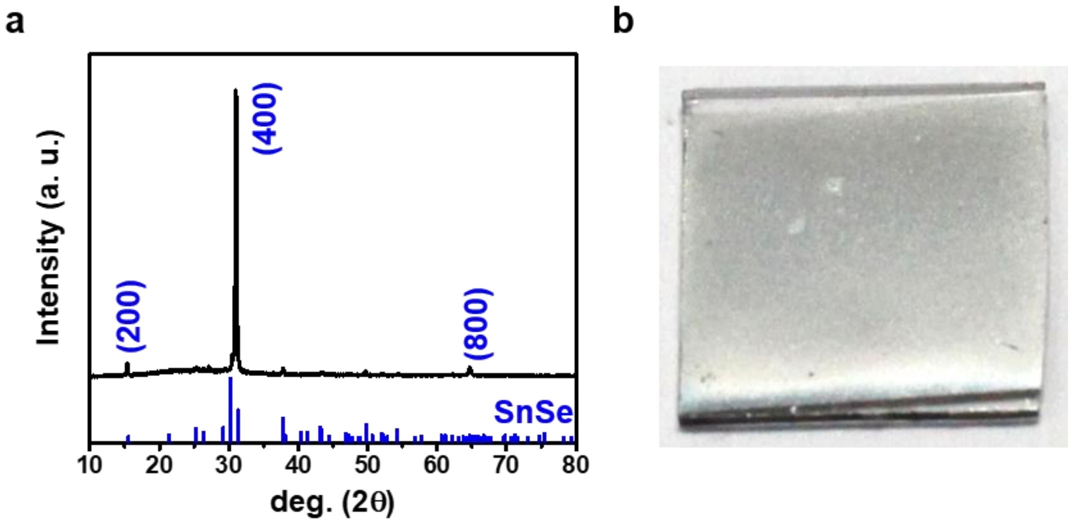

Supplementary Fig. 13 | The SnSe thin film fabricated through spray coating method. a. The XRD pattern of the SnSe thin film shows clear peaks indexed to the (200), (400), and (800) planes, confirmed its textured structure. **b**. Photograph of thin film shows even surface of thin film.

Supplementary Tables

Heat-treatment temperature	Sn:Se atomic ratio
300 ^{o}C	1:1.57
350 ^{o}C	1:1.10
400 ^{o}C	1:0.96

Supplementary Table 1 | Sn:Se ratio of SnSe thin films with various heat treatment temperatures.

Heat-treatment temperature	Sn:Se atomic ratio
1 min	1:1.57
5 min	1:1.16
9 min	1:1.01
13 min	1:0.89

Supplementary Table 2 | Sn:Se ratio of SnSe thin films with various heat treatment at 400 °C.

Supplementary References

1 Zhao, L. D. et al. Ultralow thermal conductivity and high thermoelectric figure of merit in SnSe crystals. *Nature* **508**, 373-377 (2014).

2 Han, G. *et al.* Facile surfactant-free synthesis of p-type SnSe nanoplates with exceptional thermoelectric power factors. *Angew. Chem. Int. Ed. Engl.* **55**, 6433-6437 (2016).

3 Li, Q. *et al.* Study on the thermoelectric performance of polycrystal SnSe with Se vacancies. *J. Alloys Compd.* **745**, 513-518 (2018).

4 Feng, D. *et al.* Enhanced thermoelectric properties of SnSe polycrystals via texture control. *Phys. Chem. Chem. Phys.* **18**, 31821-31827 (2016).

5 Wei, W. *et al.* Achieving high thermoelectric figure of merit in polycrystalline SnSe via introducing Sn vacancies. *J. Am. Chem. Soc.* **140**, 499-505 (2018).

6 Fu, Y. et al. Enhanced thermoelectric performance in p-type polycrystalline SnSe benefiting from texture modulation. *J. Mater. Chem. C* **4**, 1201-1207 (2016).
